# Sara Beam, editor and translator, The Trial of Jeanne Catherine: Infanticide in Early Modern Geneva

**DOI:** 10.1093/shm/hkab076

**Published:** 2021-07-23

**Authors:** Joel F Harrington

**Affiliations:** Vanderbilt University, USA

Sara Beam’s latest book is a collection of primary sources intended for classroom use. All sixty-one documents, of varying length, centre on one particular case, the 1686 trial of a young single mother, Jeanne Catherine Thomasset, accused of poisoning her own two-year-old illegitimate daughter and the five-year-old son of the rural wet nurse who is caring for them both. It is not a typical infanticide case, which usually involved the smothering or strangling of a newborn, but Beam has located and skilfully translated an impressive variety of case-related documents that allow her to convey both the drama of the investigation and trial, and the broader legal and social contexts in which they take place. The result is an early modern kind of ‘Law and Order’ that successfully engages the reader, while making many important historical points.

Beam’s forty-page introduction provides an overview of the case, as well as essential background information on the city-state of Geneva, the early modern justice system and the crime of infanticide. We learn what we need to know in order to appreciate the events as they unfold, but, in the manner of an experienced storyteller, the author withholds enough for us to be surprised as some turns of events, guessing about the truth and ultimate outcome up until the very end. This is especially important for undergraduates, who will be attracted to the narrative elements. But Beam lets the documents do the talking, which naturally means much back-and-forth in accounts, as well as inevitable contradictions. In other words, students learn the roles of agency and contingency, as well as of social structures and cultural assumptions. There are no attempts to dumb down the material or otherwise condescend, requiring readers to pay attention but at the same time providing all the assistance necessary in analysing the texts at hand.

The case itself is initiated by the father of the dead boy, who scurries from his village to confront Jeanne Catherine in Geneva and denounce her to a town *auditeur*, who in turn promptly launches an official investigation. According to the bereaved father, it was only after a brief visit by the accused and her older male cousin, during which she gave both children some candy, that both victims became violently ill and died. During the course of the investigation we hear from neighbours, relatives, magistrates, physicians and of course the accused herself—who is interrogated nine separate times, often with torture. In that sense, the book is reminiscent of Thomas Robisheaux’s excellent *Last Witch of Langenburg*, where we are able to encounter all the perspectives and determinative factors involved in a village case of suspected poisoning. Jeanne Catherine, for instance, is both a minor noblewoman and a foreigner, factors that work both for and against her in the perceptions of her neighbours and prosecutors. Students thus learn the relative importance of gender, social status, reputation, local politics, etc. They will likely also be surprised to discover the common legal assumption of guilt by authorities, the influence of learned experts, the reliance on coerced confessions and the painstaking thoroughness of such investigations, despite the use of torture.

The sources are all fluently translated and annotated with enough information to help without becoming intrusive. I was a bit surprised that the introduction itself was not annotated, but that decision was likely based on the intended audience. This does not in any way diminish the overall success of the book and its likely popularity with both students and instructors, no doubt prompting lively and enlightening discussion. Beam has impressively combined drama and analysis, the microhistorical and the macrohistorical, providing students with many important insights about the early modern period. I recommend *The Trial of Jeanne Catherine* wholeheartedly and in fact have decided to incorporate it into my own course on crime and punishment in early modern Europe.

